# Supplementary material for: Who does what to whom? graph representations of action-predication in speech relate to psychopathological dimensions of psychosis
Source: Schizophrenia (Heidelb). 2022 Jul 5;8(1):58. doi: 10.1038/s41537-022-00263-7 (PMC9261087; doi:10.1038/s41537-022-00263-7)
Supplement: Supplementary file 1 — Supplementary Material [file 41537_2022_263_MOESM1_ESM.docx]

***Who Does What to Whom? Graph Representations of Action-Predication in Speech Relate to Psychopathological Dimensions of Psychosis***

**Amir H. Nikzad , Yan Cong, Sarah Berretta, Katrin Hänsel, Sunghye Cho, Sameer Pradhan, Leily Behbehani, Danielle DeSouza, Mark Y. Liberman, Ph.D, Sunny X. Tang, M.D.**

**Supplementary Figures and Tables**

|  | **Protocol 1**  **n = 12** | **Protocol 1 – Virtual**  **n = 61** | **Protocol 2**  **n = 105** | **Protocol 3**  **n = 27** |
| --- | --- | --- | --- | --- |
| **Age: mean years (SD)** | 25.71 (5.17) | 27.68 (4.99) | 24.06 (6.45) | 25.72 (5.33) |
| **Sex: n (%)** | Female: 5 (42%)  Male: 7 (58%) | Female: 32 (52%)  Male: 29 (48%) | Female: 72 (69%)  Male: 33 (31%) | Female: 9 (33%)  Male: 18 (67%) |
| **Gender: n (%)** | Woman: 4 (33%)  Man: 6 (50%)  Genderqueer/Gender non-conforming/non-binary/other: 2 (17%) | Woman: 31 (51%)  Man: 28 (46%)  Genderqueer/Gender non-conforming/non-binary/other: 2 (3%) | Woman: 58 (55%)  Man: 36 (34%)  Genderqueer/Gender non-conforming/non-binary/other: 11 (10%)  Prefer not to answer: 1 (1%) | Woman: 5 (19%)  Man: 18 (69%)  Genderqueer/Gender non-conforming/non-binary/other: 2 (8%)  Prefer not to answer: 1 (4%) |
| **Psychotic Spectrum Disorder: n (%)**  **Break down and comorbidities** | Total: 12 (100%)  Schizophrenia: 7 (58%)  Schizoaffective Disorder: 2 (17%)  Unspecified Psychotic Disorder: 3 (25%)  Mood disorder: 5 (42%)  Anxiety disorder: 3 (25%)  Substance use disorder: 6 (50%) | Total: 17 (28%)  Schizophrenia: 9 (53%)  Schizoaffective: 1 (6%)  Schizophreniform Disorder: 2 (12%)  Unspecified Psychosis: 4 (23.5%)  Mood disorder: 6 (35.3%)  Anxiety disorder: 2 (12%)  Substance use disorder: 6 (35%) | Total: 25 (24%)  Schizophrenia: 4 (16%)  Schizoaffective: 3 (12%)  Unspecified Psychosis: 7 (28%)  Mood disorder + psychotic features: 12 (48%) | Total: 27 (100%)  Schizophrenia: 16 (59%)  Schizoaffective: 4 (15%)  Schizophreniform: 3 (11%)  Unspecified Psychosis: 4 (15%)  Mood disorder: 5 (18.5%)  Substance use disorder: 5 (18.5%) |
| **Other Psychiatric Conditions: n (%)**  **Break down and comorbidities** | Total: 0 (0%) | Total: 44 (72%)  Healthy Volunteer: 37 (84%)  Anxiety disorder: 7 (16%)  Mood disorder: 2 (4.5%)  Substance use disorder: 3 (7%) | Total: 80 (76%)  Mood disorder: 76 (95%)  Anxiety disorder: 19 (24%)  Substance use disorder: 12 (15%) | Total: 0 (0%) |
| **Ascertainment** | Inpatient | Outpatient and healthy volunteers | Inpatient and outpatient | Inpatient |
| **Data Collection Method** | Recorded in-person interaction | Recorded interaction over Microsoft Teams | In-person data collection proctored with digital app | In-person data collection proctored with digital app |
| **Assessment Context** | Other assessments included semi-structured clinical interviews and self-report scales.  Scales included in this analysis: BPRS, SANS, TLC. | Other assessments included semi-structured clinical interviews and self-report scales.  Scales included in this analysis: BPRS, SANS, TLC. | Other assessments consist of self-report questionnaires.  Scales included in this analysis: TLC. | Other assessments included semi-structured clinical interviews  Scales included in this analysis: BPRS, SANS, TLC. |
| **Picture Description Stimuli** | 1. Cookie theft scene  2. Rorschach  3. TAT | 1. Cookie theft scene  2. Rorschach  3. TAT | 1. Family kitchen scene  2. Rorschach  3. TAT | 1. Lightbulb living Room scene  2. Rorschach  3. Family kitchen scene |
| **Picture Description Prompts** | Please look at this picture and describe everything that you see. | Please look at this picture and describe everything that you see. | Please tell me what's going on in this picture. | Please tell me what's going on in this picture. |
| **Open-ended Narrative Prompts** | 1. Tell me about yourself 2. How have things been going recently? | 1. Tell me about yourself  2. How have things been going recently? | 1. Tell me about yourself  2. How have you been spending your time, recently? | 1. Tell me about yourself  2. How have you spent your time recently? |

**Supplemental Table 1.** Comprehensive clinical characteristics of participants. Note: BPRS = Brief Psychiatric Rating Scale; SANS = Scale for Assessment of Negative Symptoms; TLC = Scale for the Assessment of Thought, Language and Communication; TAT = Thematic Apperception Test; SD = Standard Deviation.

| **Utterance** | **Action Relations** | | **Predication Relations** |
| --- | --- | --- | --- |
| I grew up in Washington. | {} | | {('grow', 'i')} |
| I have two siblings. | {('i', 'two'), ('i', 'sibling')} | | {('have', 'i'), ('have', 'two'), ('have', 'sibling')} |
| I like to work a lot. | {('i', 'work'), ('i', 'lot')} | | {('like', 'i'), ('like', 'work'), ('like', 'lot'), ('work', 'i')} |
| I don’t ever give myself too much time. | {('i', 'much'), ('i', 'time'), ('i', 'i')} | | {('give', 'i'), ('give', 'much'), ('give', 'time'), ('give', 'i')} |
| I like clothes. | {('i', 'clothe')} | | {('like', 'i'), ('like', 'clothe')} |
| I don’t have a lot of clothes but I wish that I did so I can dress up however I want to. | {('i', 'lot'), ('i', 'clothe'), ('i', 'that'), ('i', 'i'), ('i', 'dress'), ('i', 'however'), ('i', 'want')} | | {('have', 'i'), ('have', 'lot'), ('have', 'clothe'), ('wish', 'i'), ('wish', 'that'), ('wish', 'dress'), ('wish', 'however'), ('wish', 'want'), ('do', 'i'), ('dress', 'i'), ('want', 'i')} |
| I went to the army as soon as I graduated. | {} | | {('go', 'i'), ('graduate', 'i')} |
| But then after that then everything just went downhill because I didn’t know what to do anymore. | {('i', 'anymore')} | | {('go', 'everything'), ('know', 'i'), ('know', 'anymore'), ('do', 'i')} |
| I started hearing voices. | {('i', 'hearing'), ('i', 'voice')} | | {('start', 'i'), ('start', 'hearing'), ('start', 'voice'), ('hearing', 'i'), ('hearing', 'voice')} |
| Honestly I feel like I’m being attacked. | {('i', 'like'), ('i', 'i'), ('i', 'attack')} | | {('feel', 'i'), ('feel', 'like'), ('feel', 'attack'), ('attack', 'i')} |
| When I look in my future I don’t really see anything right now because of the voices. | {('i', 'anything')} | | {('look', 'i'), ('see', 'i'), ('see', 'anything')} |
| **Graph Representations:** | | | |
| Semantic Graph:  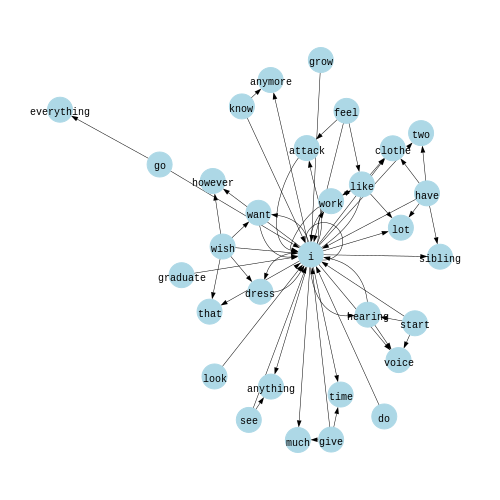 | | Structural Graph:  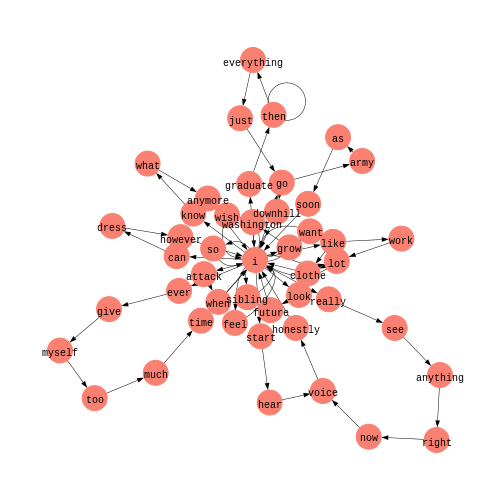 | |

**Supplemental Table 2.** Action and predication relations and structural and semantic graph representation of an open-ended narration, in response to the prompt: “Tell me about yourself”. The sample is as follows: “I grew up in Washington. I have two siblings. I like to work a lot. I don’t ever give myself too much time. I like clothes. I don’t have a lot of clothes but I wish that I did so I can dress up however I want to. I went to the army as soon as I graduated. But then after that then everything just went downhill because I didn’t know what to do anymore. I started hearing voices. Honestly I feel like I’m being attacked. When I look in my future I don’t really see anything right now because of the voices.”

| Graph Feature | Symbol | Definition | Domain |
| --- | --- | --- | --- |
| Number of Nodes | NN | Number of distinct nodes | Size |
| Number of Edges | NE | Number of distinct edges | Size |
| Diameter | Diameter | Shortest path length between the two most distant nodes | Size |
| Average Shortest Path Length | ASPL | Average number edges to pass from one randomly selected node to another | Size |
| Average Weighted Degree | AWD | Average sum of weights of edges per node | Connectedness |
| Density | Density | Number of realized edges divided by number of possible edges | Connectedness |
| Size of Largest Strongly Connected Component | LSCC | The size of largest component of the graph with all nodes being mutually reachable | Connectedness |
| The z-score of the Size of Largest Strongly Connected Component compared to 1000 random graphs | LSCCZ | z-score of LSCC in the population of 1000 randomly generated graphs of the same size (similar NN and NE) | Organization |
| The z-score of the Average Shortest Path Length compared to 1000 random graphs | ASPLZ | z-score of ASPL in the population of 1000 randomly generated graphs of the same size (similar NN and NE) | Organization |

**Supplemental Table 3.** Symbols and definitions of graph features.

| **Graph Features (n = 36)** | **Open-ended Narrative Task** | | |
| --- | --- | --- | --- |
|  | **1. Domain** | **2. Type** | **3. Task** |
| **Static Action-Predication Graph**  **Size (n = 4)** | S_AP NE,  S_AP Diameter,  S_AP ASPL | S_AP Diameter,  S_AP AWD,  S_AP Density,  D_AP Diameter,  D_AP Density,  D_AP LSCC,  D_AP ASPLZ | S_AP Diameter,  S_AP Density,  D_AP Diameter,  D_AP Density,  D_AP LSCC,  D_AP ASPLZ,  S_SEQ AWD,  S_SEQ LSCCZ,  D_SEQ NE,  D_SEQ LSCCZ,  D_SEQ ASPLZ |
| **Static Action-Predication Graph**  **Connectedness (n = 3)** | S_AP AWD,  S_AP Density,  S_AP LSCC |  |  |
| **Static Action-Predication Graph**  **Organization (n = 2)** | S_AP LSCCZ |  |  |
| **Dynamic Action-Predication Graph**  **Size (n = 4)** | D_AP NN,  D_AP Diameter,  D_AP ASPL |  |  |
| **Dynamic Action-Predication Graph**  **Connectedness (n = 3)** | D_AP AWD,  D_AP Density,  D_AP LSCC |  |  |
| **Dynamic Action-Predication Graph**  **Organization (n = 2)** | D_AP ASPLZ |  |  |
| **Static Sequential Graph**  **Size (n = 4)** | S_SEQ NE,  S_SEQ ASPL | S_SEQ NE,  S_SEQ AWD,  S_SEQ Density,  S_SEQ LSCCZ,  D_SEQ NE,  D_SEQ LSCCZ,  D_SEQ ASPLZ |  |
| **Static Sequential Graph**  **Connectedness (n = 3)** | S_SEQ AWD,  S_SEQ Density,  S_SEQ LSCC |  |  |
| **Static Sequential Graph**  **Organization (n = 2)** | S_SEQ LSCCZ,  S_SEQ ASPLZ |  |  |
| **Dynamic Sequential Graph**  **Size (n = 4)** | D_SEQ NE,  D_SEQ ASPL |  |  |
| **Dynamic Sequential Graph**  **Connectedness (n = 3)** | D_SEQ AWD,  D_SEQ Density,  D_SEQ LSCC |  |  |
| **Dynamic Sequential Graph**  **Organization (n = 2)** | D_SEQ LSCCZ,  D_SEQ ASPLZ |  |  |

**Supplemental Table 4. Survived Graph Features in Sequential VIF Comparisons in Open-ended Narrative Task.** Columns within each segment accommodate survived graph features. VIF comparison was conducted and features of highest VIF were excluded successively until a set of features all showing VIF < 5 was attained. Survived features were then passed to the next column on right for another comparison on a more integrated level. 1. Domain column shows results of intra-domain comparisons. 2. Type column presents the features integrated on graph-type level, i.e. semantic vs structural graph features. 3. Task column combines all graph features per each task. For dynamic semantic graph features belonging to three domains of size, connectedness and organization remained in the final set. Graph features of different methods are color coded. Note: S_AP = static action-predication graph feature; D_AP = dynamic action-predication graph feature; S_SEQ = static sequential graph feature; D_SEQ = dynamic sequential graph feature; NN = number of nodes; NE = number of edges; Diameter = graph diameter; ASPL = average shortest path length; AWD = average weighted degree; Density = graph density; LSCC = size of largest strongly connected component; LSCCZ = z-score of LSCC compared to 1000 random graphs; ASPLZ = z-score of ASPL compared to 1000 random graphs. More details on graph features are available in Supplemental Table 2.

| **Graph Feature** | **Task** | **Static Action-Predication Graph (S_AP)** | **Dynamic Action-Predication Graph (D_AP)** | **Static Sequential Graph (S_Seq)** | **Dynamic Sequential Graph (D_Seq)** |
| --- | --- | --- | --- | --- | --- |
| Size | | | | | |
| Number of Nodes (NN) | Picture | 0.20* | 0.21* | 0.22* | 0.30** |
|  | Open-Ended | 0.16 | 0.28** | 0.15 | 0.33*** |
| Number of Edges (NE) | Picture | 0.20* | 0.23* | 0.19* | 0.20* |
|  | Open-Ended | 0.14 | 0.26** | 0.12 | **0.36***** |
| Diameter | Picture | 0.13 | 0.13 | 0.27** | 0.32*** |
|  | Open-Ended | 0.01 | 0.10 | 0.30** | 0.28** |
| Average Shortest Path Length (ASPL) | Picture | 0.11 | 0.10 | 0.29** | 0.29** |
|  | Open-Ended | 0.10 | 0.21* | 0.30** | 0.28** |
| Connectedness | | | | | |
| Average Weighted Degree (AWD) | Picture | 0.11 | 0.12 | -0.04 | -0.30** |
|  | Open-Ended | 0.01 | 0.11 | -0.13 | -0.31** |
| Density | Picture | -0.11 | -0.16 | -0.27** | -0.30** |
|  | Open-Ended | -0.20* | -0.27** | -0.23* | -0.32*** |
| Size of Largest Strongly Connected Component (LSCC) | Picture | 0.12 | 0.15 | 0.22* | 0.11 |
|  | Open-Ended | 0.10 | 0.17 | 0.16 | 0.30** |
| Organization | | | | | |
| LSCC z-score (LSCCZ) | Picture | -0.19* | -0.24** | 0.29** | 0.20* |
|  | Open-Ended | -0.14 | -0.21* | 0.27** | 0.31** |
| ASPL z-score (ASPLZ) | Picture | -0.19* | -0.21* | 0.01 | 0.27** |
|  | Open-Ended | -0.15 | -0.23* | 0.01 | 0.27** |

A) Subset of participants matched for sex (n=152)

B) Subset of participants matched for race (n=144)

| **Graph Feature** | **Task** | **Static Action-Predication Graph (S_AP)** | **Dynamic Action-Predication Graph (D_AP)** | **Static Sequential Graph (S_Seq)** | **Dynamic Sequential Graph (D_Seq)** |
| --- | --- | --- | --- | --- | --- |
| Size | | | | | |
| Number of Nodes (NN) | Picture | 0.20* | 0.21* | 0.24* | 0.28** |
|  | Open-Ended | 0.15 | 0.23* | 0.15 | 0.32** |
| Number of Edges (NE) | Picture | 0.21* | 0.26** | 0.21* | 0.18 |
|  | Open-Ended | 0.14 | 0.21* | 0.12 | 0.31** |
| Diameter | Picture | 0.22* | 0.22* | 0.25** | 0.28** |
|  | Open-Ended | -0.4 | 0.04 | 0.28** | 0.28** |
| Average Shortest Path Length (ASPL) | Picture | 0.16 | 0.17 | 0.26** | 0.24* |
|  | Open-Ended | 0.05 | 0.15 | 0.28** | 0.29** |
| Connectedness | | | | | |
| Average Weighted Degree (AWD) | Picture | 0.16 | 0.17 | -0.01 | -0.25* |
|  | Open-Ended | 0.00 | 0.10 | -0.14 | -0.27** |
| Density | Picture | -0.09 | -0.08 | -0.31** | -0.27** |
|  | Open-Ended | -0.21* | -0.22* | -0.21* | -0.30** |
| Size of Largest Strongly Connected Component (LSCC) | Picture | 0.20* | 0.23* | 0.22* | 0.05 |
|  | Open-Ended | 0.07 | 0.12 | 0.15 | 0.22* |
| Organization | | | | | |
| LSCC z-score (LSCCZ) | Picture | -0.23* | -0.27** | 0.21* | 0.14 |
|  | Open-Ended | -0.13 | -0.16 | 0.25** | 0.29** |
| ASPL z-score (ASPLZ) | Picture | -0.22* | -0.29** | -0.05 | 0.21* |
|  | Open-Ended | -0.14 | -0.18 | 0.03 | 0.27** |

**Supplemental Table 5. Relationships between Structural and Semantic Graph Features and Psychosis in two subsamples matched for sex (A) and race (B).** Graph features are categorized into three domains of size, connectedness and psychosis. For each feature rank biserial correlation coefficient (RBC) is reported for picture description task (Picture) and open-ended narrative (Open-Ended) as a measure of effect size. The significant associations are tagged by asterisks (*=<0.05, **=<0.01, and ***<0.001). Associations survived in Bonferroni correction are bolded (alpha = 0.0003). Despite decreased effect sizes and levels of significance, the results are consistent with findings in the whole sample as presented in the Table 3.

| A) Picture description task  **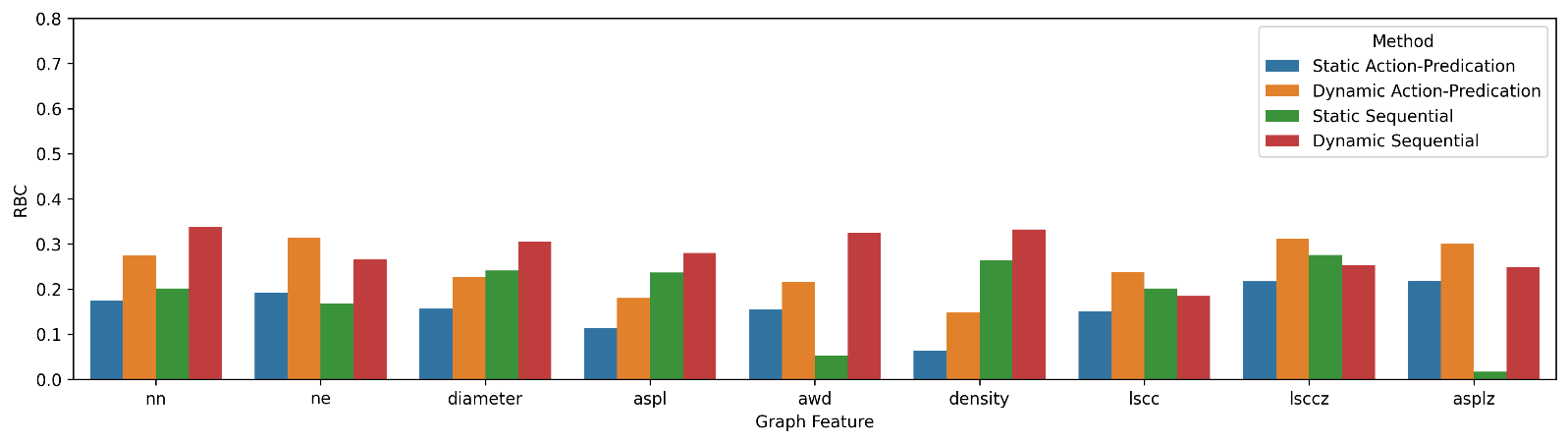** |
| --- |
|  |
| B) Open-ended narrative task  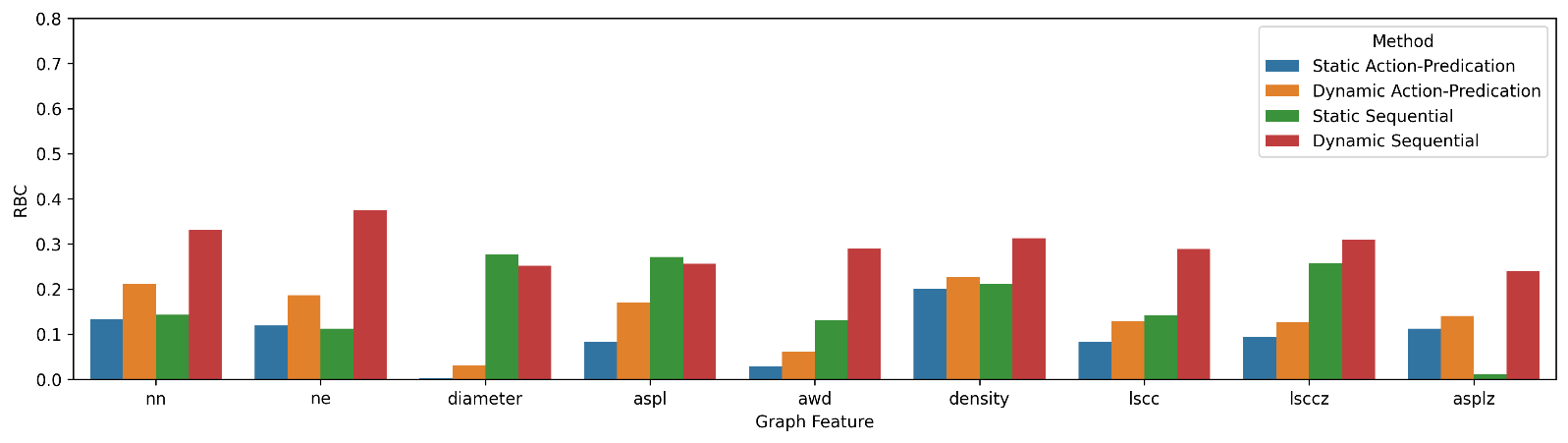 |
|  |

**Supplemental Figure 1.** Rank biserial coefficients (RBC) for the associations between speech graph features and psychosis. Different methods of measurements are marked with different colors. Dynamic measurement improved the performances of both structural and semantic speech graphs in indication of psychosis. Picture description task was produced more informative speech graph features in terms of their relationships to psychosis.

| A) Speech Disorganization |
| --- |
| **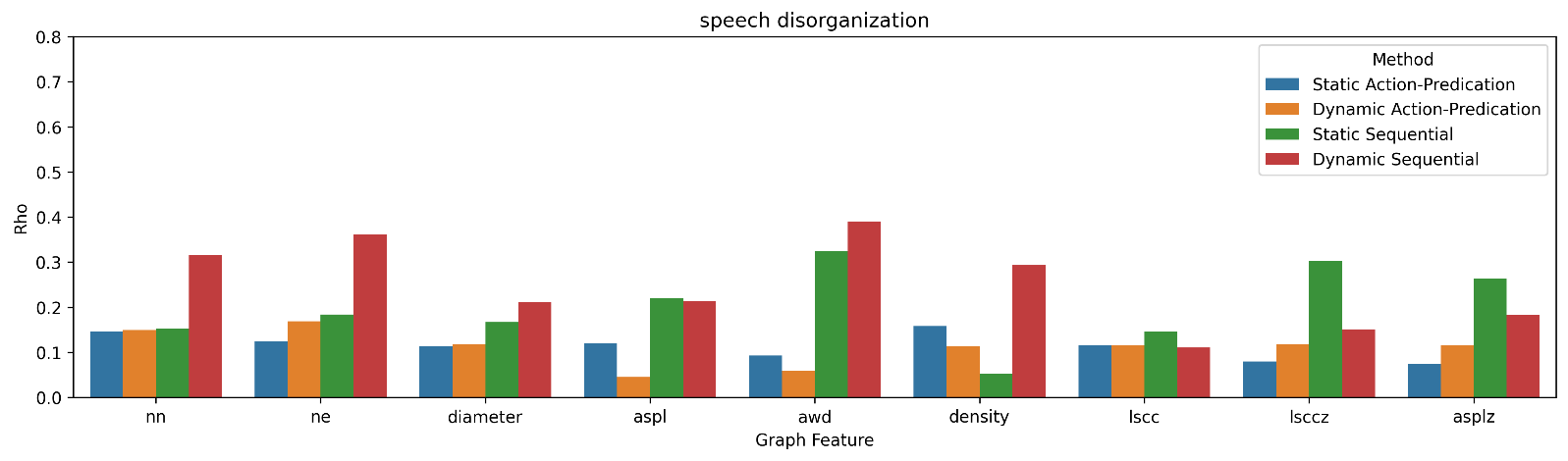** |
| B) Speech Poverty |
| **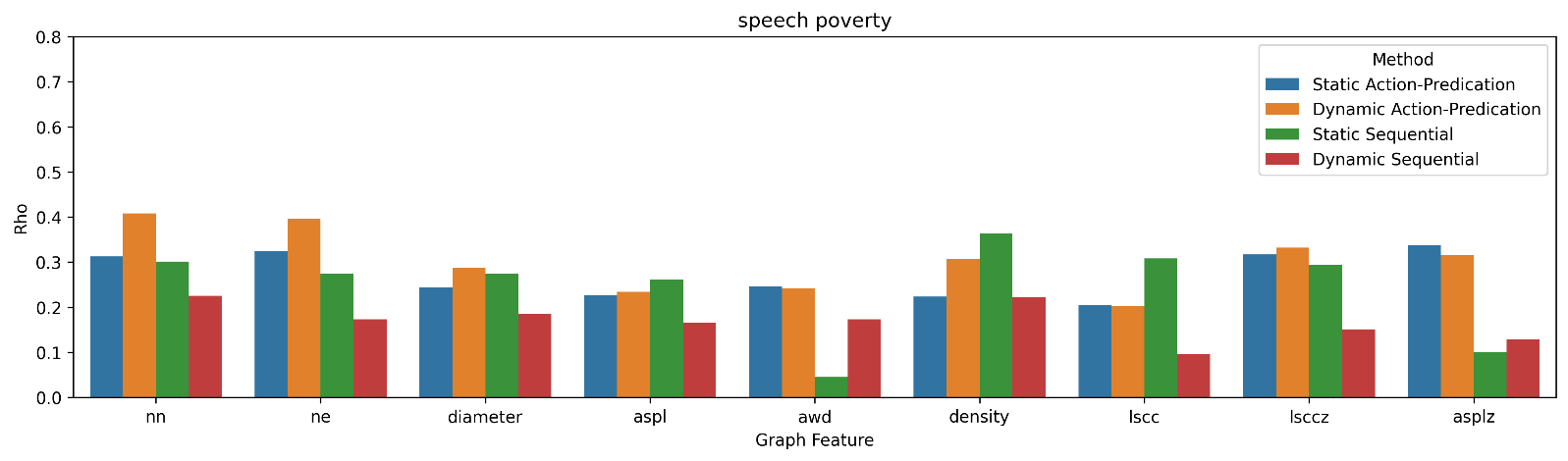** |
| C) Total BPRS Score |
| **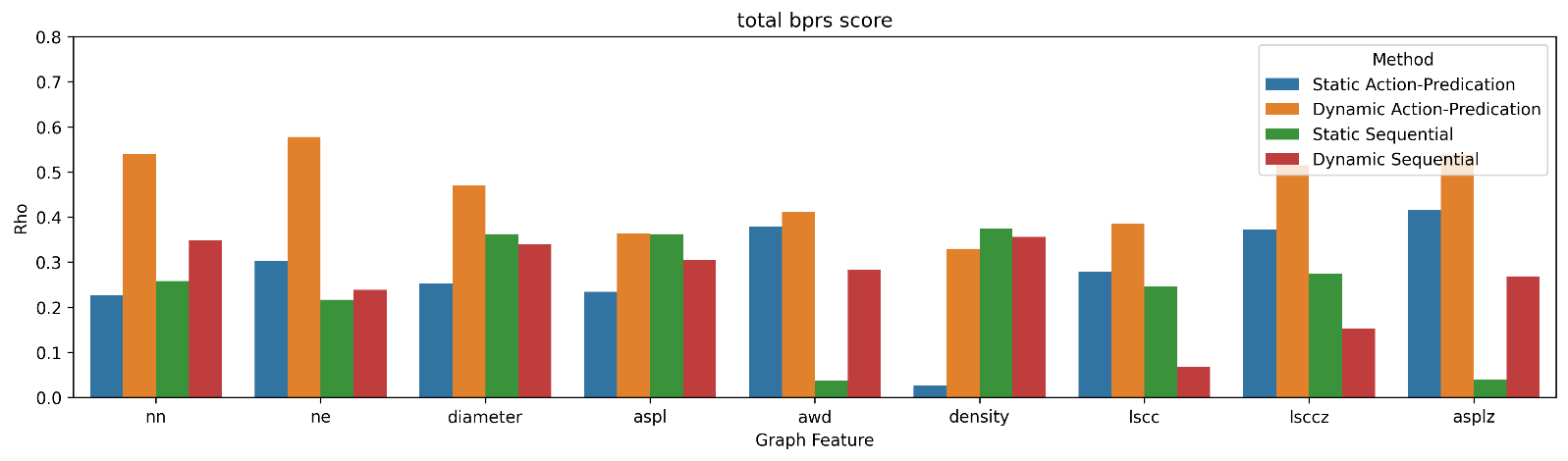** |
| D) Anxiety/Depression |
| **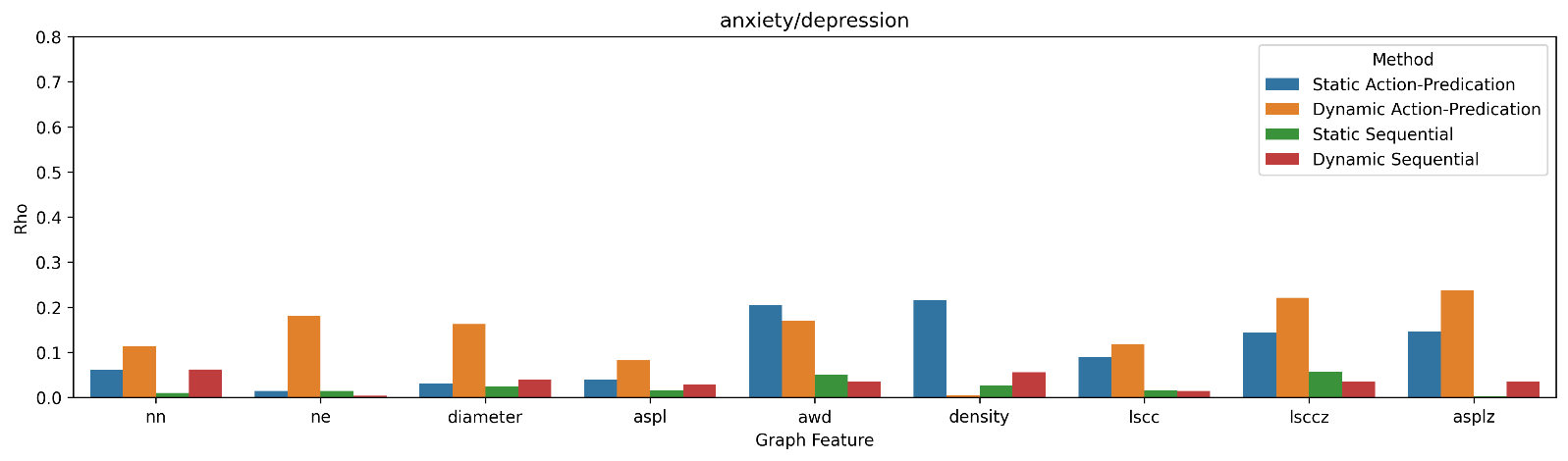** |
| E) Hostility Suspiciousness |
| **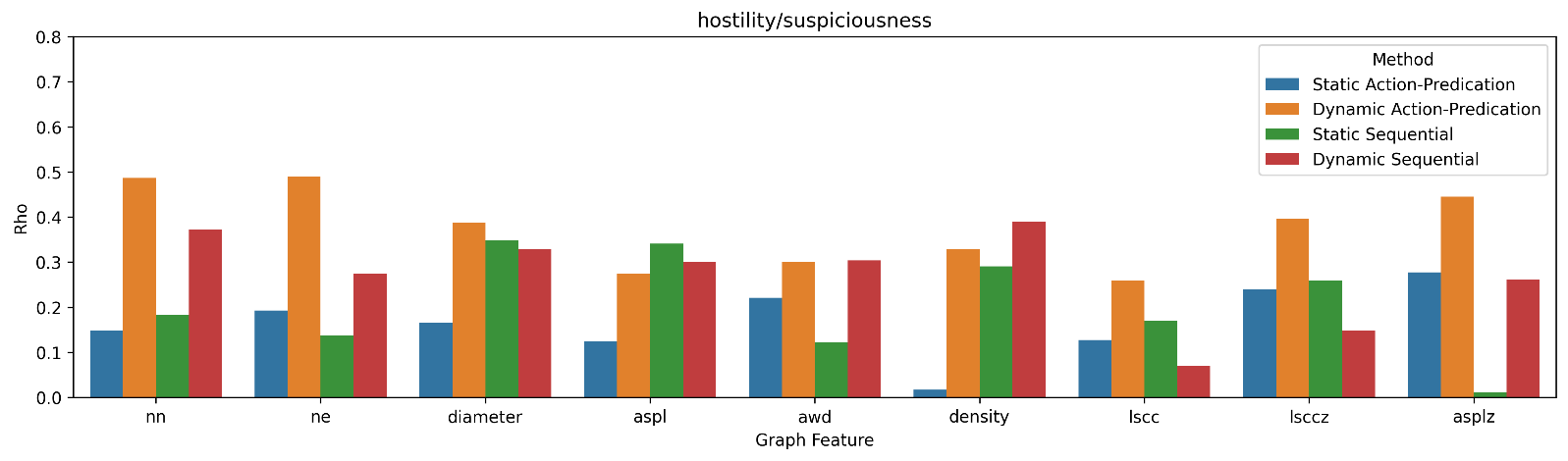** |
| F) Thought Disturbance |
| **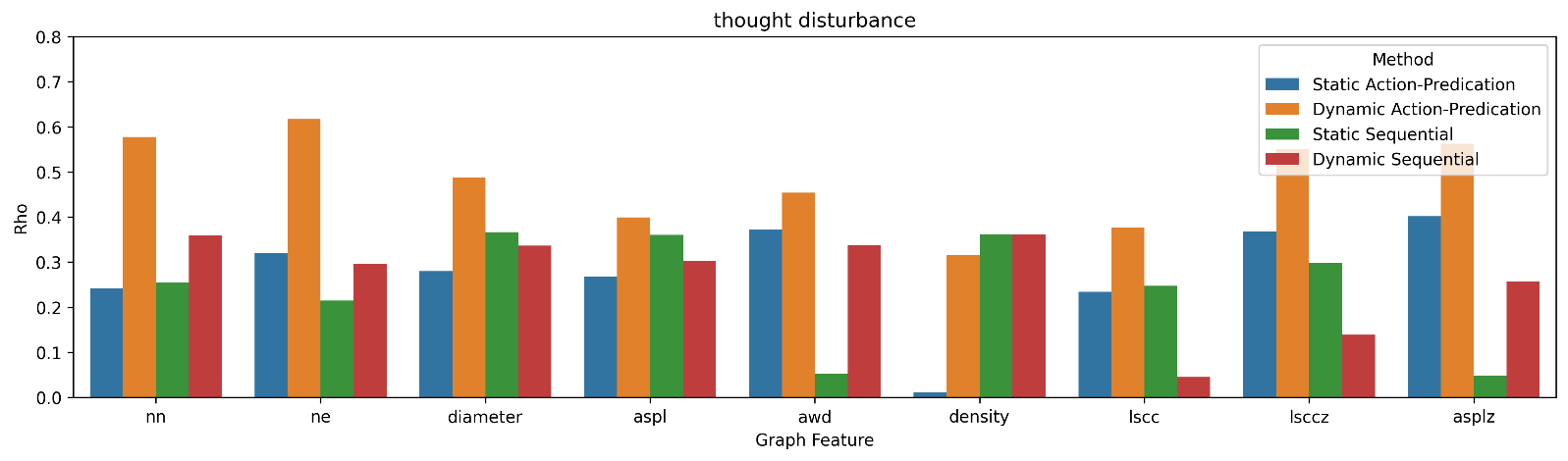** |
| G) Withdrawal/Retardation  **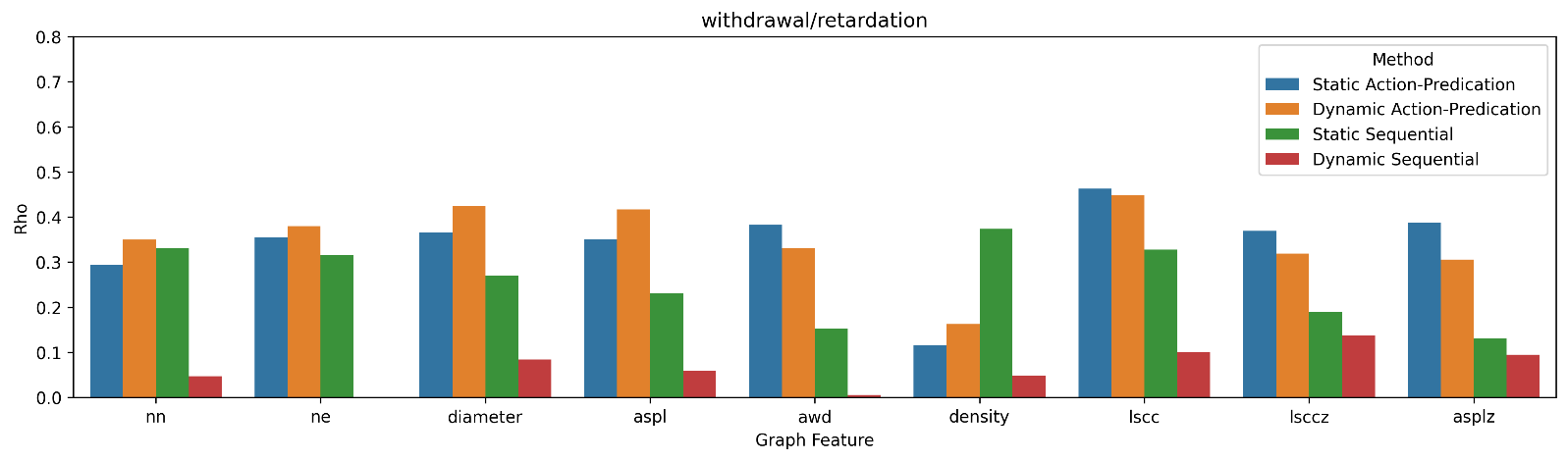** |
|  |
| H) Asocialtiy/Anhedonia |
| **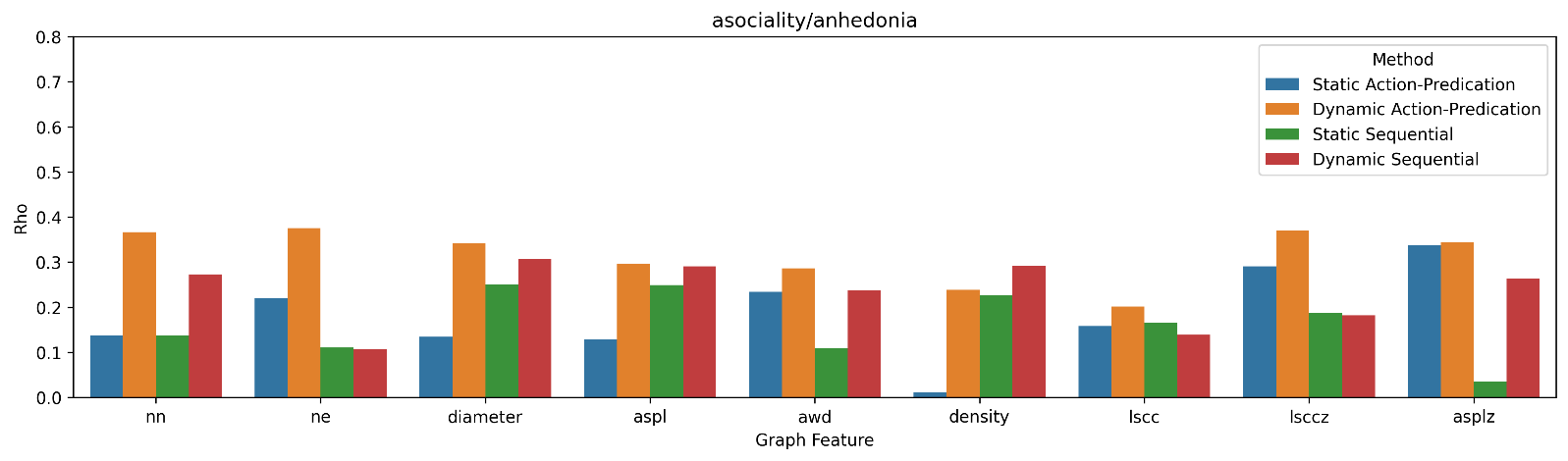** |
| I) Affect Flattening  **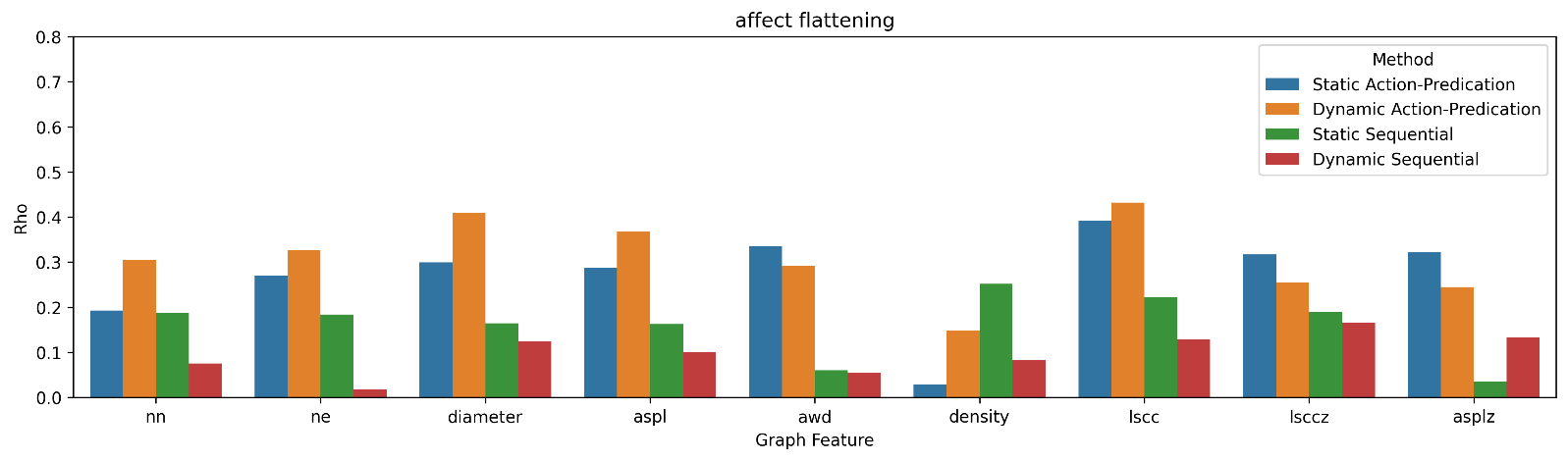** |
|  |
| J) Global Alogia |
| **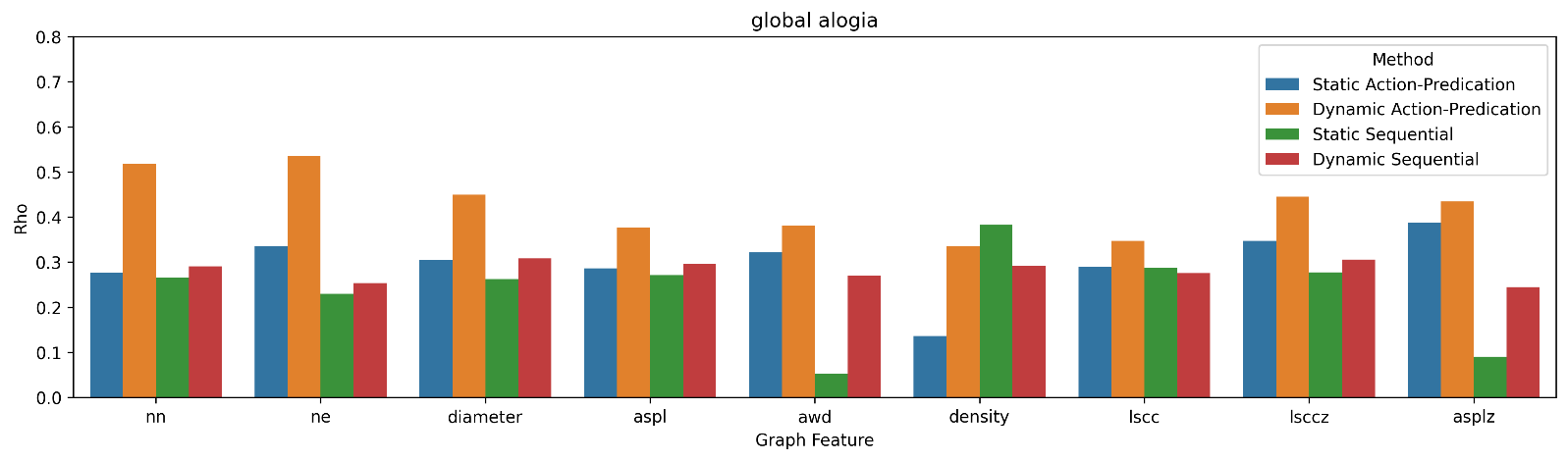** |
| K) Avolition |
| **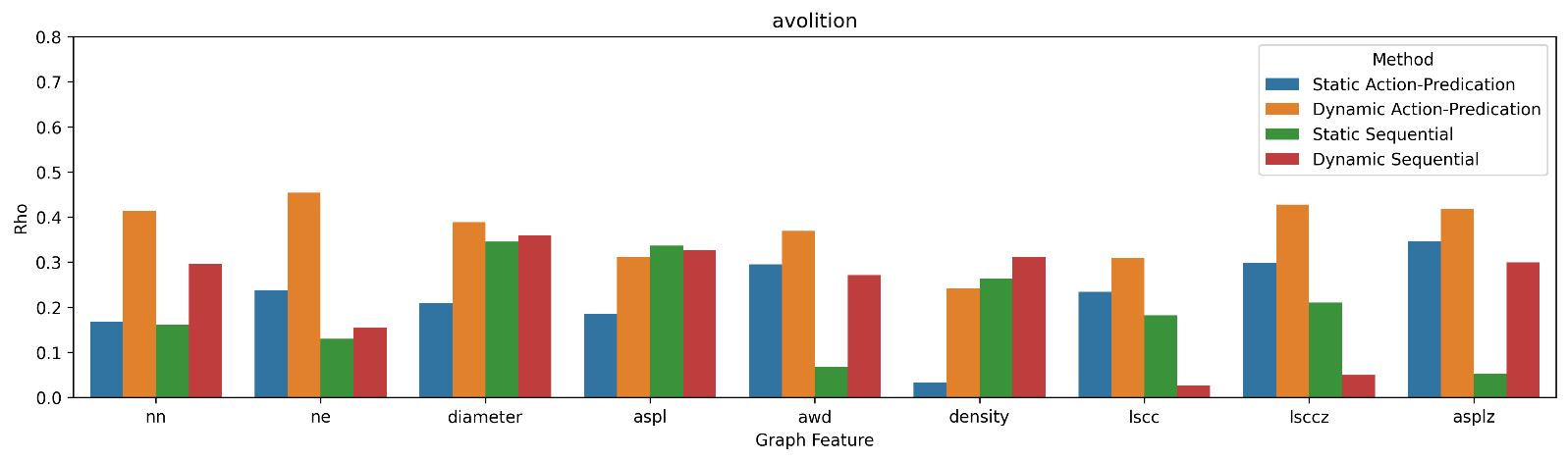** |

**Supplemental Figure 2.** Rho coefficients for the spearman correlations between speech graph features in picture description task and dimensions of psychosis. Different methods of measurements are marked with different colors.
